# Short-term effects of air pollution on the infectious disease spectrum in Shanghai, China: a time-series analysis from 2013 to 2019

**DOI:** 10.3389/fpubh.2025.1454809

**Published:** 2025-01-31

**Authors:** Yihan Lin, Hao Meng, Yong He, Wenzhuo Liang, Yiran Niu, Zhenliang Liu, Ziying Wang, Yangyang Tian, Shiyang Chang

**Affiliations:** ^1^Department of Histology and Embryology, College of Basic Medical, Hebei Medical University, Shijiazhuang, China; ^2^Department of Pathogenic Biology, College of Basic Medicine, Hebei Medical University, Shijiazhuang, China

**Keywords:** infectious diseases, air pollution, fine particulate matter, ozone, time-series study, distributed lag model

## Abstract

**Background:**

Epidemiological evidence on the effects of air pollution on infectious diseases remains inconsistent, highlighting the need for further research and analysis. We aimed to investigate the relationship between exposure to fine particulate matter (PM_2.5_) and ozone (O_3_) and the risk of national notifiable infectious diseases in Shanghai, a megacity in China.

**Methods:**

A double-pollutant model was used for each air pollutant, utilizing time-series analysis to separately apply single and distributed lag models (DLMs) to assess the exposure-lag-response relationship for 43 national notifiable infectious diseases (NNIDs) from 2013 to 2019. The model was adjusted for seasonality, long-term trends, mean temperature, relative humidity, and other air pollutants. Analysis was further conducted for seven NNID categories (vaccine-preventable; bacterial; gastrointestinal and enterovirus; sexually transmitted and bloodborne; vector-borne; zoonotic; and quarantinable diseases) as well as specific diseases.

**Results:**

The study included 661,267 NNID cases and found that PM_2.5_ and O_3_ exposures were associated with increased NNID risks, although not within the same categories. A 10 μg/m^3^ increase in O_3_ was associated with a higher risk of total NNIDs (relative risk [RR] at lag 1 month: 1.29, 95% confidence interval [CI]: 1.02–1.65), vaccine-preventable diseases (RR at lag 1 month: 1.75, 95% CI: 1.02–3.01), and sexually transmitted and bloodborne diseases (RR at lag 2 month: 1.12, 95% CI: 1.00–1.26). However, the association with PM_2.5_ remained inconclusive.

**Conclusion:**

These findings suggest a potential link between ambient air pollution exposure and the risk of infectious diseases, highlighting the urgent need for a comprehensive understanding of the relationship between air pollution and notifiable infectious diseases, as well as an in-depth evaluation of disparities across the disease spectrum.

## Introduction

China has substantially reduced the disease burden through effective infectious disease control measures (1). However, this has also led to an underestimation of infectious diseases as major causes of morbidity and mortality (1, 2). In recent years, outbreaks of emerging infectious diseases, such as COVID-19 and monkeypox, have posed new challenges to public health and driven greater interest in understanding the risk factors associated with these infectious diseases (3, 4). While recent outbreaks have drawn heightened attention to the role of weather and air pollutants in infectious diseases (5–8), assessments of the impact on a wide range of infectious diseases remain limited (3).

Ambient air pollutants, including fine particulate matter (PM_2.5_) and ozone (O_3_), may contribute to increased concentrations of bacteria, viruses, or other pathogens in the atmosphere (9). Additionally, they could act as immunosuppressive agents, weakening the human body’s typical immune defenses (3). Existing epidemiological evidence linking air pollution to infectious diseases remains limited and inconclusive, making it challenging to draw reliable conclusions from current research. For example, most studies have focused on conjunctivitis, tuberculosis (TB), and respiratory infectious diseases (especially COVID-19), yet their findings have been inconsistent (3, 10, 11), potentially due to variations in study areas, study periods and modeling specifications. Further investigations into other infectious diseases, such as diarrheal diseases, malaria, measles, hand-foot-mouth disease, mumps, and others, are needed (12–15). To the best of our knowledge, no previous study has simultaneously examined the impact of ambient air pollutants on various infectious diseases during the same study period under a unified study protocol (3, 16). Therefore, the successive infectious disease surveillance system in China presents an opportunity to provide a complete picture of the association between air pollution and various notifiable infectious diseases, as well as a comprehensive evaluation of disparities across the spectrum of diseases.

This study aimed to comprehensively examine the short-term effects of air pollution on a wide range of notifiable infectious diseases and evaluate disparities in associations across specific categories in Shanghai between 2013 and 2019. The identification of potential disparities in infectious disease burdens due to air pollution would provide direction for the targeted implementation of prevention and control measures. This includes strengthening surveillance systems to monitor specific infectious diseases or categories that may exhibit elevated risks under higher pollution levels, enabling timely interventions and optimized allocation of medical resources. Additionally, enhancing public education on the health impacts of air pollution raises awareness and encourages the adoption of protective measures, thereby mitigating associated health risks.

## Materials and methods

### Study design and infectious disease data

The study applies a time-series analysis using secondary data collected in Shanghai, a megacity in China, between 2013 and 2019. Ethical approval was not required as the research utilized secondary data that did not include any personal or identifiable information.

Monthly National Notifiable Infectious Diseases (NNIDs) data were obtained from a surveillance system. Details of this systematic, long-term infectious disease surveillance system are provided in Supplementary material. A total of 43 NNIDs were included in this study, grouped into seven categories following the previous categorization approach (17). Specifically, (I) vaccine-preventable diseases (11 diseases): includes seasonal influenza, rubella, pertussis, mumps, measles, hepatitis A, B, and D, neonatal tetanus, poliomyelitis, and diphtheria. (II) Bacteria (4 diseases) Includes tuberculosis, scarlet fever, meningococcal meningitis, and leprosy. (III) Gastrointestinal and enterovirus diseases (5 diseases): Consist of diseases primarily affecting the gastrointestinal system, such as typhoid and paratyphoid, infectious diarrhea, hand, foot, and mouth disease (HFMD), dysentery, and acute hemorrhagic conjunctivitis. (IV) Sexually transmitted and bloodborne diseases (4 diseases): Include syphilis, gonorrhea, HIV/AIDS, and hepatitis C. (V) Vector-borne diseases (7 diseases): Includes typhus, schistosomiasis, malaria, kala-azar, Japanese encephalitis, dengue, and filariasis. (VI) Zoonotic diseases (9 diseases): Include brucellosis, hepatitis E, hydatid disease, rabies, anthrax, leptospirosis, H5N1, H7N9, and severe acute respiratory syndrome (SARS). (VII) Quarantinable diseases (3 diseases): Includes hemorrhagic fever, cholera, and plague.

### Air pollutants and weather variables

The two air pollutants analyzed in this study are fine particulate matter (PM_2.5_) and ozone (O_3_), in units of μg/m^3^, in accordance with the China ambient air quality standards (GB3095-2012). Monthly average PM_2.5_ concentrations at the surface level were obtained from a nationwide dataset with a spatial resolution of 10 km (18, 19). This air pollution dataset is publicly accessible and was developed as part of the Tracking Air Pollution (TAP) project in China.^1^ It integrates multisource-fusion data and employs machine learning algorithms to improve the accuracy and reliability of the exposure data. The methodology for predicting PM_2.5_ concentrations has been described in previous studies (18, 20). In brief, a comprehensive dataset was first assembled, incorporating ground-level PM_2.5_ measurements from monitoring stations, satellite-derived aerosol optical depth (AOD), meteorological variables, land use features, population density, and elevation details, along with outputs from the Weather Research and Forecasting/Community Multiscale Air Quality Modeling System (WRF/CMAQ). This information was harmonized into a unified 10 km grid. Subsequently, PM_2.5_ concentrations in the TAP products were predicted using a two-stage machine learning approach that employed a synthetic minority oversampling technique and a tree-based gap-filling method. The cross-validation of the prediction model ranged from 0.80 to 0.88, suggesting a good simulation performance of the predictions relative to the measurements (19).

Predictions for the maximum 8-h average O_3_ concentrations were derived from the TAP dataset, utilizing a three-stage random forest-based modeling approach (21). This model integrates a wide range of data sources, including ground-level observations, CMAQ simulations, Ozone Monitoring Instrument (OMI) satellite O_3_ profiles (PROFOZ), meteorological data from MERRA-2, MODIS-derived Normalized Difference Vegetation Index (NDVI), and annual night light data from the National Centers for Environmental Information (NCEI). In the first stage, two separate sets of O_3_ predictions were generated: one incorporating satellite data and the other excluding it, addressing gaps due to missing satellite retrievals. The non-satellite model ensured full spatial coverage. The second stage employed an elastic-net regression to combine predictions from both models, producing a unified set of O_3_ estimates. In the final stage, residuals—calculated as the difference between observed maximum 8-h O_3_ concentrations and second-stage predictions—were modeled using kriging interpolation to capture spatiotemporal variations. These residuals were then added to the second-stage estimates to produce the final predictions. The model’s performance, assessed using 5-fold cross-validation, showed an R^2^ value of 0.70, indicating good agreement with ground-level measurements.

Monthly meteorological data from 2013 and 2019, including average temperature (°C) and relative humidity (%), were obtained from the National Meteorological Data Sharing Center.^2^

### Statistical analysis

To investigate the short-term effect of air pollutants on NNIDs, a time series regression (TSR) using a generalized linear model was applied (22). TSR is widely used in environmental epidemiology for assessing short-term associations, defined in this study as the relationship between monthly variations in air pollutant exposure and changes in NNID counts. Socioeconomic and demographic factors are assumed to remain relatively stable over neighboring months. A Quasi-Poisson model was selected to account for the overdispersion in NNIDs counts. Various time adjustment methods were explored to account for long-term trends and seasonal patterns in NNIDs, including linear trends, time interactions, Fourier terms, and different splines with varying degrees of freedom (df). Among these, a natural cubic B-spline (NCS) with 8 df per year was identified as the most appropriate for our analysis (Supplementary Figure S1).

The generalized linear regression model has been extensively applied in previous global TSR studies examining the short-term health effects of air pollutants (23–26). This study used two distinct modeling approaches: the single lag model and the Distributed Lag Model (DLM). These approaches were chosen to capture the typically linear and delayed health effects of air pollutant exposure (27, 28). The single lag model assumes that a unit increase in pollutants is associated with an outcome at a specific future time point, such as a 1-month lag representing the relationship between exposure during the previous month and outcomes in the current month. In contrast, the DLM accounts for cumulative exposure effects over multiple lag months, thereby capturing the impact of past exposures distributed over several months on current health outcomes. Specifically, the DLM incorporates a cross-basis function, which simultaneously models the exposure and lag dimensions under the assumptions of linear relationships with NNIDs (29–31). For non-infectious diseases, a maximum lag of 21 days is commonly applied, which approximates lag1 in this study (31). However, given the more complex causal pathways associated with infectious diseases and recommendations from previous literature highlighting the extended duration of infectious immune periods (22, 32), a lag period of up to 2 months was considered.

To control for time-varying confounders, a double-pollutant model was applied for each pollutant, adjusting for the influence of the other pollutant as well as time-varying weather variables, including mean temperature and relative humidity (29). The adjustment for the confounder was performed using an NCS with 3 df applied to the moving average of each covariate over the lag period, as suggested by previous research (25, 33, 34). Additionally, a stratified analysis was conducted using NNID categories and specific causes. To ensure sufficient statistical power, only subgroups with a sample size greater than 5,000 were included in the stratification analysis (35). The effect estimates are reported as relative risks (RRs) with 95% confidence intervals (CIs), representing the change in risk per 10 μg/m^3^ increase in PM_2.5_ or O_3_ at each lag month.

### Sensitivity analysis

To assess the robustness of the analysis, several sensitivity analyses were conducted. First, the lag period was extended to 3 months to examine a wider range of relationship patterns and lag durations. Second, single-pollutant models were fitted for comparison with the results from double-pollutant models. All the analyses were performed in R software (version 4.2.1; https://www.rproject.org/) with the “*dlnm*” package. A two-sided *p*-value of less than 0.05 was considered statistically significant.

## Results

### Characteristics of NNIDs and air pollutants

Between January 2013 and December 2019, a total of 661,267 incident cases of NNIDs were reported in Shanghai (Table 1). The majority of reported cases were gastrointestinal and enterovirus diseases (351,464 cases, 53%), followed by sexually transmitted and bloodborne diseases (137,036 cases, 20%), vaccine-preventable diseases (93,134 cases, 14%), and bacterial diseases (73,851, 11%). In contrast, fewer cases were reported for vector-borne diseases (447 cases), zoonotic diseases (5,300 cases), and quarantinable diseases (35 cases). Time-series plots revealed evident seasonal patterns across all NNIDs categories, along with an overall declining trend over time, except for vector-borne and zoonotic diseases (Supplementary Figure S2).

**Table 1:** Summary statistics of 43 notifiable infectious diseases by category and specific diseases during 2013–2019 in Shanghai.

	n	Mean^a^	SD^a^		n	Mean^a^	SD^a^
Vaccine-preventable diseases	93,134	1109.0	1538.0	Vector-borne diseases	447	5.3	4.8
SI	63,728	759.0	1550.0	Typhus	-	-	-
Rubella	1,433	17.1	37.3	Schistosomiasis	-	-	-
Pertussis	353	4.20	6.1	Malaria	220	2.6	1.7
Mumps	18,361	219.0	109.0	Kala-azar	-	-	-
Measles	2,612	31.1	55.9	JE	9	0.1	0.3
Hepatitis A	1,795	21.4	9.7	Dengue	218	2.6	4.5
Hepatitis B	4,847	57.7	25.2	Filariasis	-	-	-
Hepatitis D^b^	5	0.1	0.3	Zoonotic diseases	5,300	63.1	23.8
NT	-	-	-	Brucellosis	35	0.4	0.7
Poliomyelitis	-	-	-	Hepatitis E	5,225	62.2	23.8
Diphtheria	-	-	-	HD	6	0.1	0.3
Bacterial diseases	73,851	879.0	248.0	Rabies	12	0.1	0.4
TB	48,338	575.0	95.4	Anthrax	-	-	-
SF	25,475	303.0	231.0	Leptospirosis	-	-	-
MM	16	0.2	0.4	H5N1	-	-	-
Leprosy	22	0.3	0.7	H7N9^c^	22	0.3	1.0
Gastrointestinal and enterovirus diseases	351,464	4,184.0	2743.0	SARS	-	-	-
T/P	186	2.2	1.7	Quarantinable diseases	35	0.4	1.1
ID	41,589	495.0	214.0	HF	29	0.3	1.1
HFMD	308,266	3670.0	2714.0	Cholera	6	0.1	0.3
Dysentery	1,241	14.8	12.9	Plague	-	-	-
AHC	182	2.2	3.1	Total^d^	661,267	7,872.2	2,837.9
Sexually transmitted and bloodborne diseases	137,036	1,631.0	272.0				
Syphilis	94,688	1,127.0	165.0				
Gonorrhea	37,937	452.0	136.0				
AIDS	3,889	46.3	18.3				
Hepatitis C	522	6.2	4.5				

SI, Seasonal influenza; NT, Neonatal tetanus; TB, Tuberculosis; SF, Scarlet fever; MM, Meningococcal meningitis; T/P, Typhoid and paratyphoid; ID, Infectious diarrhea; HFMD, Hand, foot, and mouth disease; AHC, Acute hemorrhagic conjunctivitis; AIDS, Acquired immune deficiency syndrome; JE, Japanese encephalitis; HD, Hydatid disease; SARS, Severe acute respiratory syndrome. HF, Hemorrhagic fever. -: no cases.

a Average and standard deviation (SD) of monthly cases during 2013–2019.

b Available from 2016-1.

c Available from 2013-12.

d Total numbers during 2013–2019.

During the study period, the average monthly concentrations of PM_2.5_ and O_3_ were 46.8 μg/m^3^ and 124.1 μg/m^3^, respectively (Table 2). The corresponding monthly ambient weather variables were 17.4°C for mean temperature and 72.8% for relative humidity. Seasonal variations were evident for all weather variables (Supplementary Figure S3), and a notable decreasing trend in PM_2.5_ levels was observed over time. Pairwise correlation analysis indicated moderate correlations between air pollutants and meteorological variables (Supplementary Figure S4).

**Table 2:** Summary statistics for monthly levels of air pollutants and weather variables in Shanghai from 2013 to 2019.

	PM_2.5_ (μg/m^3^)	O_3_ (μg/m^3^)	Temperature (°C)	Relative humidity (%)
Minimum	17.4	60.1	4.3	57.0
10th	25.7	74.4	6.1	65.0
25th	33.0	98.5	10.1	68.8
Median	45.1	130.6	18.2	74.0
Mean	46.8	124.1	17.4	72.8
SD	19.6	33.3	8.3	5.9
75th	56.5	152.5	24.2	77.0
90th	74.5	161.2	28.3	80.0
Maximum	118.4	180.8	32.0	83.0

th, percentile of the distribution; SD, standard deviation.

### Short-term effects of air pollutants on NNIDs

The short-term effects of PM_2.5_ and O_3_ on NNIDs were assessed using the single lag model and DLMs, with the results summarized in Table 3.

**Table 3:** Relative risks (and 95% confidence intervals) for the monthly infectious diseases per unit increase in air pollutant concentrations were estimated using the double-pollutant model.

		Total	Vaccine-preventable	Bacteria	Gastrointestinal and enterovirus	Sexually transmitted and bloodborne	Zoonotic
PM_2.5_	Single Lag Model						
	Lag0	**1.09 (1.00, 1.19)**	**1.22 (1.01, 1.47)**	1.01 (0.92, 1.11)	1.13 (1.00, 1.29)	1.02 (0.96, 1.08)	1.00 (0.87, 1.15)
	Lag1	0.90 (0.79, 1.02)	0.77 (0.59, 1.02)	1.01 (0.88, 1.15)	0.85 (0.71, 1.01)	1.00 (0.92, 1.09)	1.02 (0.85, 1.24)
	Lag2	0.99 (0.87, 1.11)	1.07 (0.84, 1.36)	0.93 (0.84, 1.04)	0.98 (0.82, 1.16)	0.94 (0.88, 1.00)	0.95 (0.81, 1.11)
	Distributed Lag Model						
	Lag0	0.92 (0.65, 1.29)	1.47 (0.70, 3.11)	0.78 (0.57, 1.07)	0.76 (0.48, 1.19)	**0.82 (0.70, 0.97)**	0.89 (0.53, 1.47)
	Lag1	0.75 (0.43, 1.30)	1.37 (0.39, 4.78)	0.65 (0.39, 1.09)	0.51 (0.25, 1.06)	**0.71 (0.54, 0.92)**	0.81 (0.35, 1.89)
	Lag2	0.86 (0.64, 1.16)	1.28 (0.66, 2.47)	0.75 (0.57, 1.00)	0.71 (0.48, 1.05)	**0.79 (0.68, 0.91)**	0.85 (0.54, 1.34)
	Net effect^1^	0.59 (0.18, 1.89)	2.58 (0.19, 35.07)	0.38 (0.13, 1.14)	0.28 (0.06, 1.28)	**0.46 (0.26, 0.81)**	0.61 (0.10, 3.57)
O_3_	Single Lag Model						
	Lag0	0.91 (0.85, 0.98)	0.84 (0.72, 0.97)	1.00 (0.91, 1.09)	0.94 (0.85, 1.03)	0.98 (0.94, 1.03)	0.97 (0.87, 1.08)
	Lag1	1.05 (0.99, 1.12)	**1.20 (1.06, 1.36)**	1.01 (0.93, 1.09)	1.05 (0.97, 1.13)	1.01 (0.97, 1.06)	1.02 (0.94, 1.12)
	Lag2	1.00 (0.95, 1.06)	0.93 (0.82, 1.05)	1.01 (0.95, 1.08)	1.00 (0.93, 1.07)	1.01 (0.97, 1.05)	1.00 (0.92, 1.07)
	Distributed Lag Model						
	Lag0	1.09 (0.91, 1.30)	1.25 (0.84, 1.86)	1.22 (0.97, 1.55)	1.11 (0.85, 1.43)	1.11 (0.97, 1.27)	1.06 (0.77, 1.45)
	Lag1	**1.29 (1.02, 1.65)**	**1.75 (1.02, 3.01)**	1.36 (0.98, 1.88)	1.28 (0.90, 1.82)	1.19 (0.99, 1.43)	1.13 (0.73, 1.76)
	Lag2	**1.18 (1.01, 1.38)**	1.29 (0.94, 2.23)	1.22 (0.99, 1.50)	1.16 (0.92, 1.47)	**1.12 (1.00, 1.26)**	1.08 (0.82, 1.42)
	Net effect^1^	1.67 (0.95, 2.93)	2.84 (0.83, 9.75)	2.03 (0.96, 4.28)	1.64 (0.72, 3.75)	1.48 (0.97, 2.25)	1.29 (0.47, 3.53)

The model was adjusted for seasonality and long-term trend, mean temperature, relative humidity, and O_3_ or PM_2.5_. Bold estimates indicate *p* < 0.05.

1 Cumulative risk per 10 μg/m^3^ change in each air pollutant.

**Figure 1:**
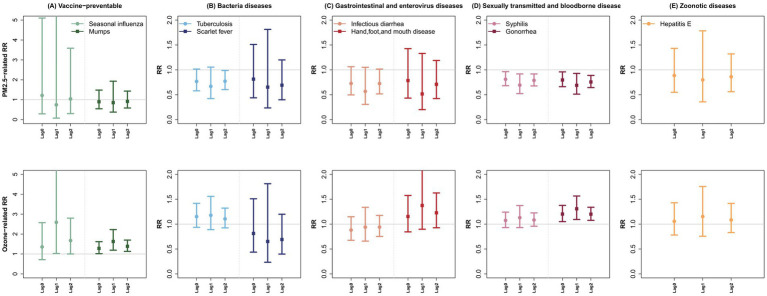
Relative risks (and 95% confidence intervals) of infectious diseases associated with a 10 μg/m^3^ increase in PM_2.5_ and O_3_ for each NNID category **(A-E)**. The double-pollutant distributed lag model (DLM) was adjusted for seasonality, long-term trends, co-pollutants, mean temperature, and relative humidity.

In the single lag analysis, where the effects of other lag periods were not adjusted for, PM_2.5_ exposure in the current month (lag 0) was significantly associated with an increased risk of total NNIDs (RR: 1.09, 95% CI: 1.00–1.19) and vaccine-preventable diseases (RR: 1.22, 95% CI: 1.01–1.47). However, findings from the DLMs indicated that most associations between PM_2.5_ exposure and NNID categories across individual lags were not statistically significant. Notably, PM_2.5_ exposure at all lag months, as well as cumulative PM_2.5_ exposure, was associated with a decreased risk of sexually transmitted and bloodborne diseases (Table 3). Consistent patterns in risk estimates were observed across specific NNID diseases within the category and over their respective lag periods (Figure 1). For example, for specific sexually transmitted and bloodborne diseases, a 10-μg/m^3^ increase in PM_2.5_ over lag 0–2 was associated with a 56% (95% CI: −76– −20) and 59% (95% CI: −79– −22) decrease in monthly syphilis and gonorrhea cases, respectively (Supplementary Table S1). In addition, lower PM_2.5_ exposure at lag 2 was linked to a higher risk of tuberculosis (RR: 0.77, 95%CI: 0.60–0.99).

For O_3_, significant associations with total NNIDs were found at lag 1 and 2 in the DLMs, although these associations were not observed in the single lag models. Specifically, each 10-μg/m^3^ increase in O_3_ at lag 1 and 2 was associated with a 29% (95% CI: 2–65) and 18% (95% CI: 1–38) risk increase in monthly total NNID cases, respectively. For specific categories, higher O_3_ concentrations at lag 1 were significantly associated with an increased risk of vaccine-preventable diseases in both single lag models (RR: 1.20, 95% CI: 1.06–1.36) and DLMs (RR: 1.75, 95% CI: 1.02–3.01) (Table 3). Furthermore, in the DLM, higher O_3_ exposure at lag 2 was linked to an elevated risk of sexually transmitted and bloodborne diseases (RR: 1.12, 95% CI: 1.00–1.26). These associations remained consistent for specific diseases within the category, with effect sizes generally larger in the DLMs compared to the single lag models, particularly for diseases such as seasonal influenza, mumps, scarlet fever, and gonorrhea (Figure 1, Supplementary Table S1).

### Sensitivity analysis

The sensitivity analyses indicated that the associations remained generally robust, with or without adjustment for the other pollutants (Supplementary Table S2). When a longer lag period of up to 3 months was applied, the results from the DLMs exhibited inconsistencies across lag durations and NNIDs categories for PM_2.5_ exposure. For O_3_, the observed associations weakened as the lag periods increased (Supplementary Table S3).

## Discussion

This study extensively assesses the relationship between ambient air pollution and a broad range of notifiable infectious diseases utilizing the infectious disease surveillance system. It provides a comprehensive and detailed evaluation of risk disparities across various infectious diseases. Our findings suggest potential associations between exposures to PM_2.5_ and O_3_ and total NNIDs, with variations in the associations observed across specific categories and diseases depending on the pollutant.

Evidence on the relationship between air pollution and infectious diseases remains limited, often focusing on individual diseases, which hinders comparisons across studies. In our study, we found no adverse effects of PM_2.5_ or O_3_ on tuberculosis (TB), which is consistent with recent meta-analyses (3, 11). However, a previous literature review reported contrasting evidence, suggesting a positive relationship between PM_2.5_ exposure and TB (10). Further large-scale studies encompassing a broader range of infectious diseases are needed to achieve a more comprehensive understanding and comparison of the effects of air pollution on infectious diseases.

The mechanisms underlying the adverse health effects of air pollution on infectious diseases are poorly understood. The observed associations may be explained by the hypothesis that air pollutants can enhance the presence of bacteria, viruses, or other pathogens in the ambient air. This effect may arise from specific components in urban PM_2.5_, chemical reactions of air pollutants (such as alterations in pH levels and heavy metals), as well as meteorological factors like temperature and humidity (36–38). Moreover, ambient air pollutants may exert an immunosuppressive effect, potentially compromising the immune system in the human body and thus impacting human health (39, 40). Interestingly, our results indicate that PM_2.5_ exposure had a more immediate effect on NNIDs, with the peak observed in the current month, while O_3_ exposure showed a delayed effect and peaked at a one-month lag. We hypothesize that the effects of these pollutants may vary depending on the stage of infection or through mechanisms beyond inflammation and oxidative stress. However, further investigation is required. The sensitivity analysis with a prolonged lag period suggests that the observed impacts were sensitive to the choice of lag period. Overall, we observed greater inconsistency in the estimates with longer lag periods (2 and 3 months of lag).

Our study has several strengths. First, it included over 6 million infectious disease cases from 43 causes over 7 years in a large urban setting, providing high statistical power and enhancing the generalizability of our findings to other urban populations with similar climates. Second, we employed flexible modeling methods to capture the complex exposure-lag-response relationship, as air pollutants often display delayed effects. This approach also allowed for the consideration and adjustment of time-varying confounders. Third, in the absence of a universally adopted classification for infectious diseases, we categorized NNIDs into seven categories based on previous research, facilitating a comprehensive examination of the relationships between air pollution and a broad spectrum of NNIDs. This categorization enabled detailed comparisons and assessments of disparities across diseases, offering critical insights that could inform targeted interventions and further research into the distinct pathogenic pathways underlying these diseases.

This study has several limitations that should be interpreted with caution. First, we assumed that all incident NNID cases were exposed to the same monthly averaged levels of air pollutants and weather based on city-level data. Such measurements may not fully capture spatial variations in exposure across urban and suburban areas within this large city. Second, this time-series analysis used monthly averages, which may influence the adequacy of the statistical power and the results. Nonetheless, in single-city studies using Poisson regression, the total number of counts and the variation in exposure are the dominant factors determining model power and assessment precision, rather than time resolution or duration of study period (35). Third, we could not explore associations between air pollution and certain categories of NNIDs, such as vector-borne diseases, due to a limited sample size (<5,000). Further studies should focus on identifying associations between specific diseases within each category, with larger sample sizes and more precise resolution. Fourth, we did not address the potential issue of multicollinearity arising from immune population dynamics and the strong autocorrelation in disease transmission (22). Finally, due to the lack of data, the analysis did not include other pollutants such as nitrogen dioxide, sulfur dioxide, and carbon monoxide, as well as some time-varying confounders, including changes in behavior and public health policies.

## Conclusion

In conclusion, our study found an association between ambient air pollution and infectious diseases, revealing significant disparities across different disease categories. These findings have important public health implications, emphasizing the need for targeted preventive and control strategies for infectious diseases or categories particularly sensitive to air pollution. Moreover, the results can inform interventions and mitigation measures regarding air pollution to further reduce the health burdens of air pollution in Shanghai. By addressing a critical gap in the existing evidence, our study underscores the urgent need for comprehensive evaluation and prompt action to mitigate the substantial burden of air pollution-attributable infectious diseases.

## Data Availability

The original contributions presented in the study are included in the article/Supplementary material. Further inquiries can be directed to the corresponding author.

## Footnotes

^1^ http://tapdata.org.cn/

^2^ http://data.cma.cn/
