# Supplementary material for: Short-term effects of air pollution on the infectious disease spectrum in Shanghai, China: a time-series analysis from 2013 to 2019
Source: Front Public Health. 2025 Jan 31;13:1454809. doi: 10.3389/fpubh.2025.1454809 (PMC11825447; doi:10.3389/fpubh.2025.1454809)
Supplement: Supplementary file 1 [file Data_Sheet_1.docx]

**Supplemental Material**

**List of Contents**

**Supplemental Text**

**Figure S1.** Choices of methodology and parameter for modeling seasonality and long-time trend.

**Figure S2.** Time-series plots of different categories of infectious disease during 2013-2019.

**Figure S3.** Time-series plots of air pollutants and weather variables during 2013-2019.

**Figure S4.** Spearman correlation matrix of air pollutants and weather variables during 2013-2019.

**Table S1.** Association between monthly air pollutants and specific infectious diseases in the double-pollutant DLM model.

**Table S2**. Relative risk (and 95% CIs) of monthly number of infectious diseases per unit increase in ozone in single-pollutant model.

**Table S3.** Relative risk (and 95% CIs) of monthly number of infectious diseases per unit increase in air pollutants in the double-pollutant model.

**Supplemental Text**

The China Information System for Disease Control and Prevention (CISDCP), an internet-based infectious disease surveillance platform, was established in 2003 by the Chinese Government. This system provides comprehensive, long-term surveillance and covers over 85% of health facilities across China [1-3]. All infectious diseases are diagnosed clinically or through laboratory confirmation in accordance with guidelines issued by the National Health Commission of the People’s Republic of China (<http://www.nhc.gov.cn/>).

Hospitals, disease control and prevention centers, community health centers, township health centers, and village health stations report standardized case information to the CISDCP database within 24 hours of diagnosis through a real-time internet-based system [2]. Case data are recorded electronically by physicians responsible for confirming diagnoses or by trained personnel. Each case is documented using standardized infectious disease reporting forms, which include demographic details, case classification, date of symptom onset, and date of diagnosis [4].

The uploaded data undergo verification by local disease control center staff to check for errors, missing information, duplicate entries, and laboratory confirmation. The system includes safeguards to prevent duplicate reporting of cases by different medical facilities. For this study, only clinically or laboratory-confirmed cases were included, while suspected cases were excluded from the analysis.

[1] Wang L, Wang Y, Jin S, Wu Z, Chin DP, Koplan JP, Wilson ME (2008). Emergence and control of infectious diseases in China. Lancet 372(9649):1598-605. doi: 10.1016/S0140-6736(08)61365-3.

[2] Zhang H, Wang L, Lai S, Li Z, Sun Q, Zhang P (2017). Surveillance and early warning systems of infectious disease in China: From 2012 to 2014. Int J Health Plann Manage 32(3):329-338. doi: 10.1002/hpm.2434.

[3] Dong Y, Wang L, Burgner DP, Miller JE, Song Y, Ren X, Li Z, Xing Y, Ma J, Sawyer SM, Patton GC (2020). Infectious diseases in children and adolescents in China: analysis of national surveillance data from 2008 to 2017. BMJ 369:m1043. doi: 10.1136/bmj.m1043.

[4] Chen L, Wang L, Xing Y, Xie J, Su B, Geng M, Ren X, Zhang Y, Liu J, Ma T, Chen M, Ma Q, Jiang J, Cui M, Guo T, Yuan W, Song Y, Dong Y, Ma J (2023). Disparity in spectrum of infectious diseases between in-school and out-of-school children, adolescents, and youths in China: findings from a successive national surveillance from 2013 to 2021. Lancet Reg Health West Pac 38:100811. doi: 10.1016/j.lanwpc.2023.100811.


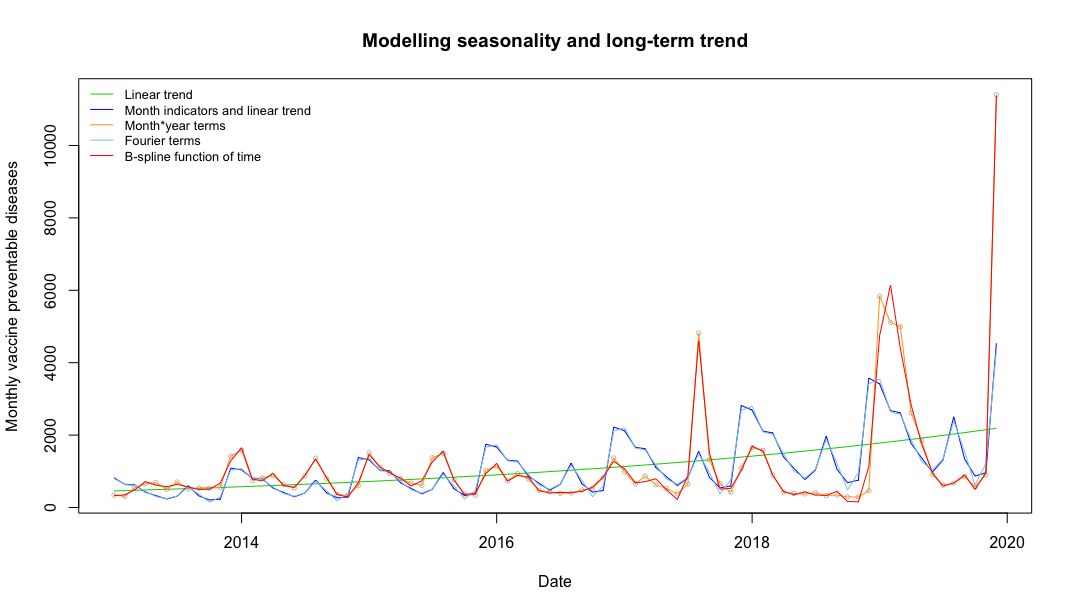

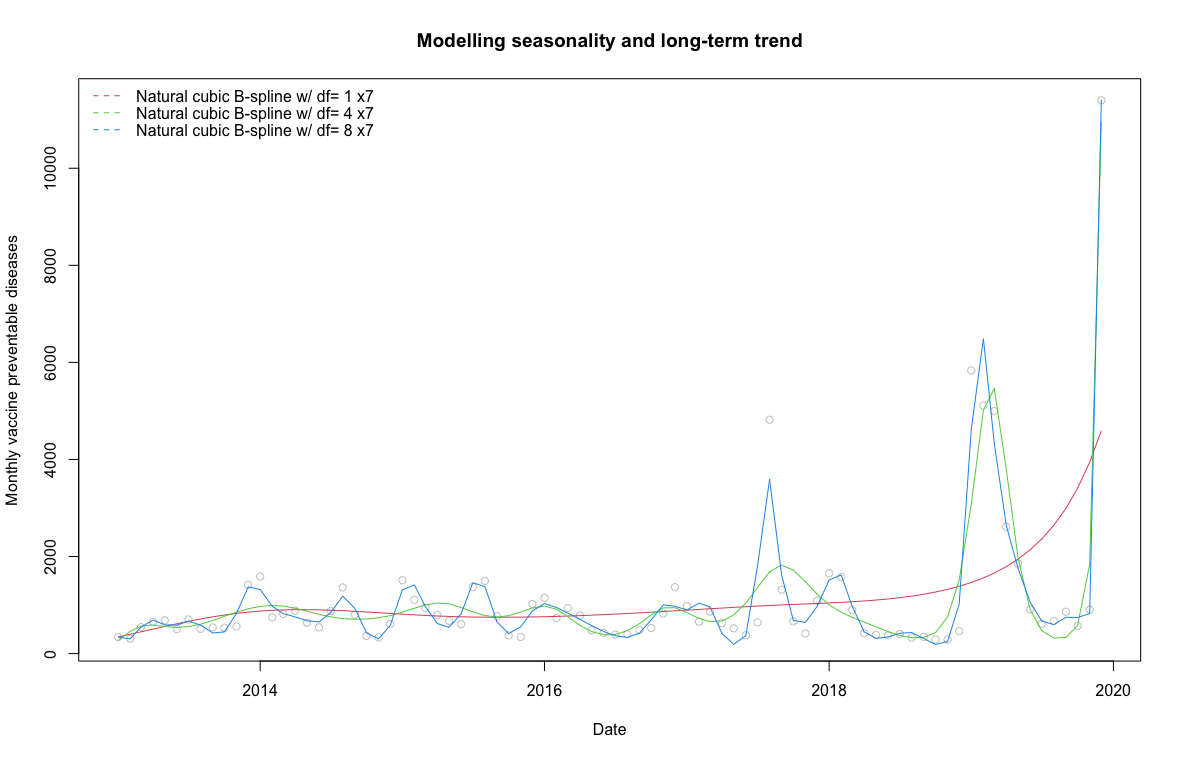
**Fig S1.** Choices of methodology and parameter for modeling seasonality and long-time trend.

*Note: The grey dots represent the observed monthly counts of infectious diseases, while the lines illustrate the time trend modeling using various methods. Vaccine preventable diseases are used as an example to present the results, with consistent modeling patterns observed across other categories.*


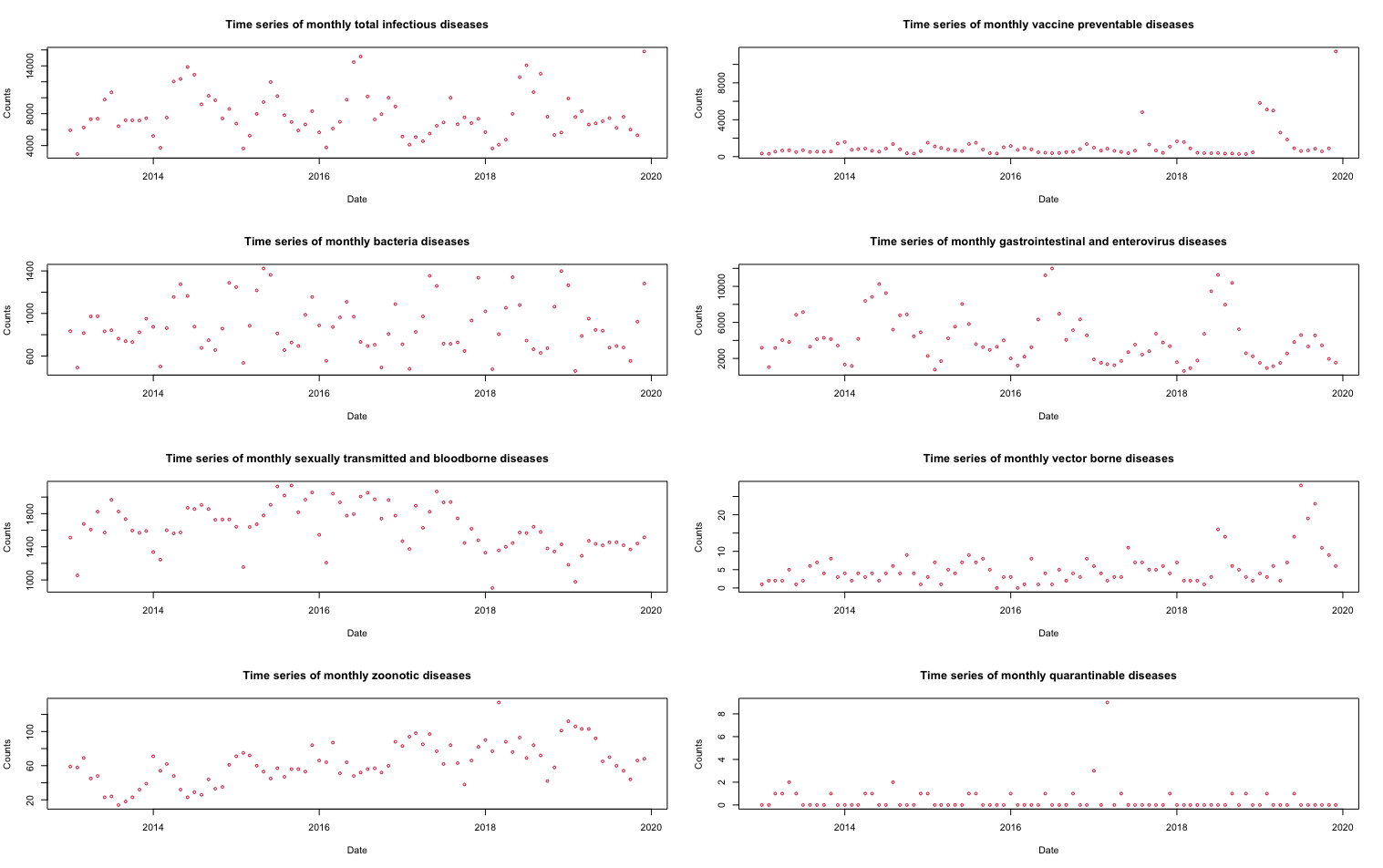
**Fig S2.** Time-series plots of different categories of infectious disease during 2013-2019.


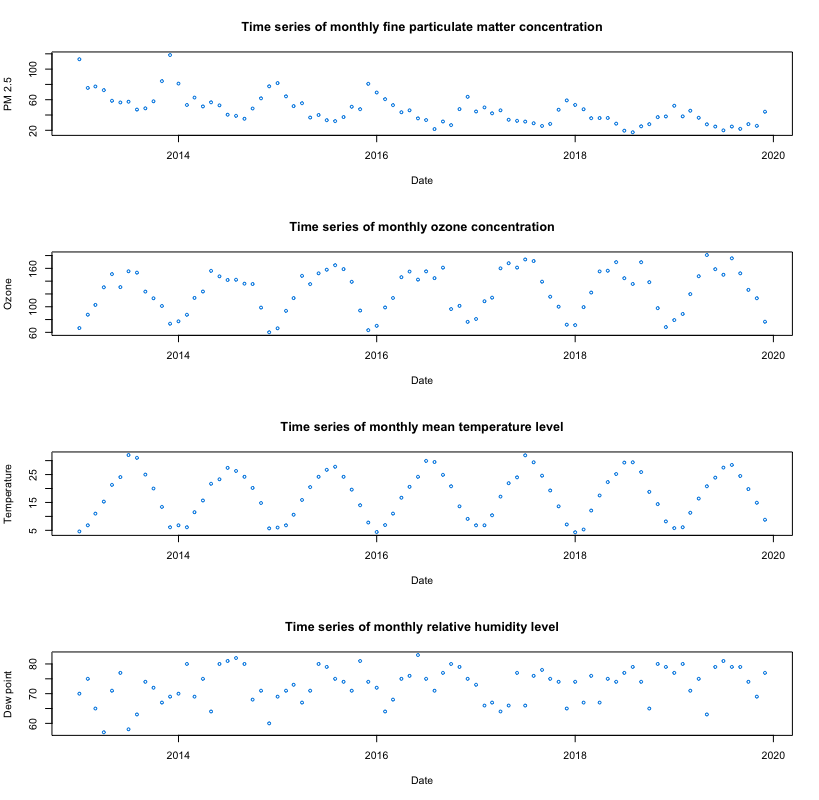


**Fig S3.** Time-series plots of air pollutants and weather variables during 2013-2019.


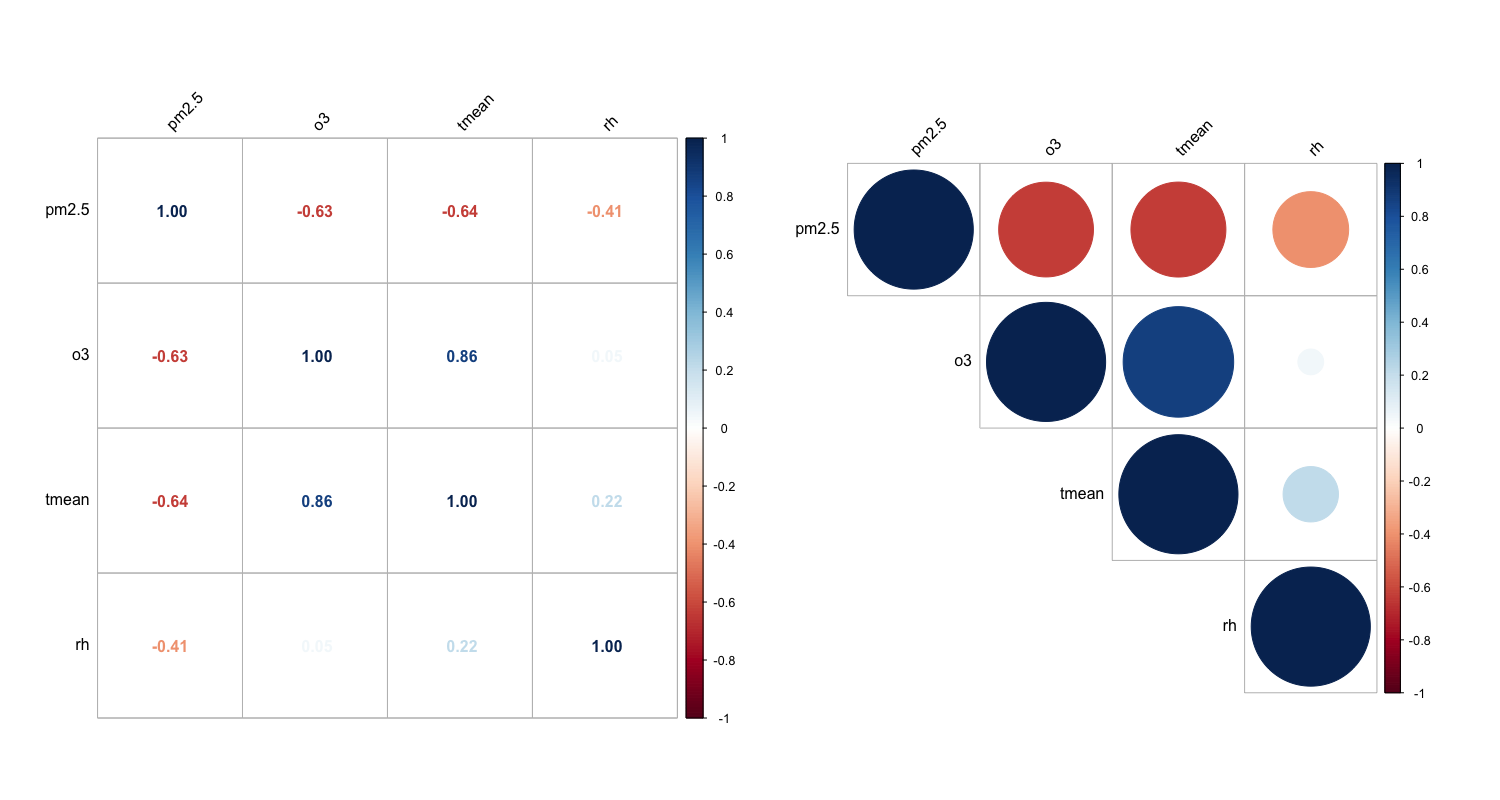
 **Fig S4.** Spearman correlation matrix of air pollutants and weather variables during 2013-2019.

| **Table S1.** Association between monthly air pollutants and specific infectious diseases in the double-pollutant DLM model. | | | | | | | | |
| --- | --- | --- | --- | --- | --- | --- | --- | --- |
|  | **PM_2.5_** | | | | **Ozone** | | | |
|  | **Lag0** | **Lag1** | **Lag2** | **Net effect** | **Lag0** | **Lag1** | **Lag2** | **Net effect** |
| **Vaccine preventable diseases** | | | | | | | | |
| SI | 1.21 (0.29, 5.10) | 0.74 (0.07, 7.49) | 1.04 (0.30, 3.59) | 0.94 (0.01, 127.91) | 1.36 (0.71, 2.58) | **2.59 (1.03, 6.55)** | **1.68 (1.00, 2.80)** | 5.89 (0.77, 44.96) |
| Mumps | 0.90 (0.54, 1.48) | 0.85 (0.38, 1.93) | 0.91 (0.58, 1.43) | 0.70 (0.12, 3.92) | **1.28 (1.02, 1.62)** | **1.63 (1.19, 2.23)** | **1.39 (1.13, 1.70)** | **2.90 (1.39, 6.02)** |
| **Bacteria diseases** | | | | | | | | |
| TB | 0.77 (0.58, 1.02) | 0.67 (0.42, 1.05) | **0.77 (0.60, 0.99)** | 0.39 (0.15, 1.04) | 1.15 (0.94, 1.42) | 1.18 (0.89, 1.56) | 1.11 (0.92, 1.32) | 1.50 (0.78, 2.88) |
| SF | 0.81 (0.44, 1.51) | 0.65 (0.23, 1.81) | 0.69 (0.40, 1.20) | 0.37 (0.04, 3.15) | **1.59 (1.10, 2.31)** | **2.15 (1.27, 3.65)** | **1.68 (1.22, 2.33)** | **5.77 ( 1.75, 19.05)** |
| **Gastrointestinal and enterovirus diseases** | | | | | | | | |
| ID | 0.73 (0.50, 1.06) | 0.57 (0.31, 1.05) | 0.72 (0.52, 1.02) | 0.30 (0.08, 1.09) | 0.88 (0.68, 1.15) | 0.94 (0.66, 1.34) | 0.94 (0.75, 1.18) | 0.78 (0.34, 1.77) |
| HFMD | 0.78 (0.43, 1.43) | 0.52 (0.20, 1.33) | 0.71 (0.42, 1.19) | 0.29 (0.04, 2.16) | 1.15 (0.84, 1.58) | 1.38 (0.90, 2.11) | 1.23 (0.93, 1.63) | 1.95 (0.72, 5.27) |
| **Sexually transmitted and bloodborne diseases** | | | | | | | | |
| Syphilis | **0.81 (0.68, 0.96)** | **0.69 (0.52, 0.92)** | **0.79 (0.68, 0.92)** | **0.44 (0.24, 0.80)** | 1.08 (0.93, 1.24) | 1.13 (0.93, 1.37) | 1.08 (0.96, 1.23) | 1.32 (0.84, 2.07) |
| Gonorrhea | **0.80 (0.66, 0.96)** | **0.69 (0.51, 0.93)** | **0.76 (0.64, 0.89)** | **0.41 (0.22, 0.78)** | **1.20 (1.05, 1.38)** | **1.31 (1.09, 1.56)** | **1.20 (1.08, 1.34)** | **1.89 (1.25, 2.86)** |
| **Zoonotic diseases** | | | | | | | | |
| Hepatitis E | 0.89 (0.55, 1.43) | 0.80 (0.36, 1.78) | 0.86 (0.56, 1.32) | 0.61 (0.11, 3.25) | 1.06 (0.78, 1.43) | 1.15 (0.76, 1.76) | 1.09 (0.83, 1.42) | 1.32 (0.50, 3.47) |
| Notes: SI: Seasonal influenza; TB: Tuberculosis; SF: Scarlet fever; ID: Infectious diarrhea; HFMD: Hand, foot, and mouth disease. | | | | | | | | |

| **Table S2**. Relative risk (and 95% CIs) of monthly number of infectious diseases per unit increase in air pollutants in single-pollutant model. | | | | | | | | | | | | |  |
| --- | --- | --- | --- | --- | --- | --- | --- | --- | --- | --- | --- | --- | --- |
|  | | **Total** | | **Vaccine preventable** | | **Bacteria** | | **Gastrointestinal and enterovirus** | **Sexually transmitted and bloodborne** | | **Zoonotic** | |  |
| **PM_2.5_** | ***Single Lag Model*** | | | | | | | | | | | | |
|  | Lag0 | **1.10 (1.02, 1.19)** | **1.29 (1.10, 1.52)** | | 1.04 (0.94, 1.14) | | 1.09 (0.98, 1.22) | | | 1.02 (0.97, 1.08) | | 1.00 (0.88, 1.15) | |
|  | Lag1 | 0.90 (0.81, 0.99) | 0.75 (0.61, 0.92) | | 0.94 (0.84, 1.06) | | 0.92 (0.81, 1.05) | | | 0.98 (0.91, 1.05) | | 1.01 (0.86, 1.19) | |
|  | Lag2 | 1.02 (0.93, 1.12) | 1.12 (0.92, 1.36) | | 0.99 (0.90, 1.10) | | 0.97 (0.86, 1.09) | | | 0.97 (0.91, 1.03) | | 0.94 (0.82, 1.07) | |
|  |  |  |  | |  | |  | | |  | |  | |
|  | ***Distributed Lag Model*** | | | | | | | | | | | | |
|  | Lag0 | 0.91 (0.70, 1.19) | 1.49 (0.83, 2.67) | | 0.75 (0.56, 1.00) | | 0.79 (0.56, 1.11) | | | **0.82 (0.71, 0.94)** | | 0.72 (0.48, 1.09) | |
|  | Lag1 | 0.72 (0.47, 1.12) | 1.31 (0.49, 3.49) | | 0.57 (0.36, 0.92) | | 0.56 (0.32, 0.99) | | | **0.68 (0.54, 0.85)** | | 0.55 (0.28, 1.09) | |
|  | Lag2 | 0.85 (0.68, 1.08) | 1.23 (0.74, 2.04) | | 0.75 (0.59, 0.97) | | 0.71 (0.52, 0.97) | | | **0.80 (0.71, 0.90)** | | 0.71 (0.50, 1.00) | |
|  | Total^1^ | 0.56 (0.23, 1.40) | 2.40 (0.32, 18.23) | | 0.38 (0.13, 1.14) | | 0.31 (0.10, 1.03) | | | **0.44 (0.28, 0.71)** | | 0.28 (0.07, 1.14) | |
|  |  | | | | | | | | | | | |  |
| **O_3_** | ***Single Lag Model*** | | | | | | | | | | | |  |
|  | Lag0 | 0.97 (0.91, 1.03) | | 0.99 (0.84, 1.16) | | 0.99 (0.93, 1.07) | | 0.95 (0.89, 1.01) | 0.98 (0.95, 1.02) | | 0.94 (0.86, 1.03) | |  |
|  | Lag1 | 1.02 (0.97, 1.08) | | 1.04 (0.90, 1.21) | | 1.01 (0.95, 1.07) | | 1.04 (0.97, 1.10) | 1.02 (0.98, 1.05) | | 1.04 (0.96, 1.13) | |  |
|  | Lag2 | 1.02 (0.97, 1.07) | | 1.00 (0.88, 1.14) | | 1.01 (0.95, 1.07) | | 1.00 (0.94, 1.07) | 1.00 (0.97, 1.04) | | 0.98 (0.91, 1.06) | |  |
|  |  |  | |  | |  | |  |  | |  | |  |
|  | ***Distributed Lag Model*** | | | | | | | | | | | |  |
|  | Lag0 | **1.16 (1.02, 1.30)** | | **1.63 (1.08, 2.45)** | | 1.17 (0.97, 1.41) | | 1.06 (0.88, 1.28) | 1.08 (0.98, 1.20) | | 0.99 (0.78, 1.27) | |  |
|  | Lag1 | **1.37 (1.12, 1.69)** | | **2.21 (1.21, 4.05)** | | 1.31 (0.99, 1.72) | | 1.22 (0.90, 1.65) | **1.18 (1.01, 1.36)** | | 1.09 (0.75, 1.58) | |  |
|  | Lag2 | **1.24 (1.09, 1.42)** | | **1.56 (1.10, 2.23)** | | 1.19 (1.00, 1.41) | | 1.15 (0.94, 1.40) | **1.11 (1.01, 1.21)** | | 1.05 (0.83, 1.33) | |  |
|  | Total^1^ | **1.98 (1.26, 3.11)** | | **5.62 (1.49, 21.20)** | | 1.82 (0.98, 3.38) | | 1.49 (0.76, 2.91) | **1.41 (1.01, 1.96)** | | 1.13 (0.50, 2.60) | |  |
| Notes: ^1^ cumulative risk per 10 µg/m^3^ change in each air pollutant. Model adjusted for seasonality and long-term trend, mean temperature, and relative humidity. | | | | | | | | | | | | |  |

| **Table S3.** Relative risk (and 95% CIs) of monthly number of infectious diseases per unit increase in air pollutants in the double-pollutant model. | | | | | | | |
| --- | --- | --- | --- | --- | --- | --- | --- |
|  |  | **Total** | **Vaccine preventable** | **Bacteria** | **Gastrointestinal and enterovirus** | **Sexually transmitted and bloodborne** | **Zoonotic** |
| **PM_2.5_** | ***Single Lag Model*** | | | | | | |
|  | Lag0 | **1.13 (1.04, 1.22)** | 1.14 (0.93, 1.39) | 1.09 (0.95, 1.24) | **1.12 (1.01, 1.25)** | 1.04 (0.98, 1.10) | 1.10 (0.96, 1.25) |
|  | Lag1 | 0.90 (0.82, 0.99) | 0.96 (0.78, 1.18) | 0.93 (0.81, 1.06) | **0.88 (0.79, 0.99**) | 0.97 (0.91, 1.03) | 0.95 (0.82, 1.09) |
|  | Lag2 | 1.02 (0.93, 1.11) | 0.96 (0.82, 1.12) | 0.99 (0.88, 1.11) | 1.04 (0.94, 1.16) | 0.99 (0.94, 1.04) | 0.98 (0.87, 1.09) |
|  | Lag3 | 1.04 (0.96, 1.12) | 1.06 (0.92, 1.20) | 1.06 (0.96, 1.18) | 1.03 (0.93, 1.14) | **1.05 (1.00, 1.09)** | 1.07 (0.98, 1.18) |
|  | ***Distributed Lag Model*** | | | | | | |
|  | Lag0 | 1.06 (0.74, 1.50) | 0.89 (0.39, 2.07) | 0.70 (0.41, 1.17) | 1.04 (0.66, 1.65) | 0.92 (0.77, 1.09) | 1.05 (0.63, 1.74) |
|  | Lag1 | 0.99 (0.46, 2.15) | 0.57 (0.10, 3.40) | 0.44 (0.14, 1.36) | 1.08 (0.39, 2.98) | 0.85 (0.58, 1.25) | 1.09 (0.37, 3.28) |
|  | Lag2 | 1.16 (0.54, 2.48) | 0.55 (0.09, 3.22) | 0.55 (0.18, 1.68) | 1.41 (0.52, 3.80) | 0.98 (0.67, 1.43) | 1.32 (0.44, 3.90) |
|  | Lag3 | 1.16 (0.81, 1.66) | 0.78 (0.34, 1.81) | 0.88 (0.52, 1.51) | 1.33 (0.83, 2.11) | 1.09 (0.92, 1.30) | 1.29 (0.77, 2.16) |
|  | Total^1^ | 1.41 (0.16, 12.43) | 0.22 (0.00, 34.56) | 0.15 (0.01, 3.62) | 2.09 (0.12, 36.02) | 0.84 (0.29, 2.46) | 1.94 (0.09, 43.27) |
|  |  | | | | | | |
| **O_3_** | ***Single Lag Model*** | | | | | | |
|  | Lag0 | 0.99 (0.92, 1.06) | 0.99 (0.85, 1.15) | 1.02 (0.92, 1.14) | 0.98 (0.90, 1.06) | 1.00 (0.95, 1.05) | 0.97 (0.88, 1.07) |
|  | Lag1 | 0.97 (0.92, 1.03) | 0.98 (0.86, 1.12) | 0.98 (0.89, 1.07) | 0.98 (0.91, 1.04) | 1.00 (0.96, 1.05) | 1.01 (0.93, 1.09) |
|  | Lag2 | **1.05 (1.00, 1.10)** | 1.04 (0.93, 1.16) | 1.01 (0.94, 1.09) | 1.05 (0.99, 1.12) | 1.00 (0.96, 1.03) | 1.00 (0.93, 1.08) |
|  | Lag3 | **0.93 (0.88, 0.99)** | 0.93 (0.83, 1.03) | 0.97 (0.89, 1.06) | 0.95 (0.88, 1.03) | 1.00 (0.96, 1.04) | 0.99 (0.91, 1.07) |
|  | ***Distributed Lag Model*** | | | | | | |
|  | Lag0 | 1.01 (0.79, 1.29) | 0.90 (0.55, 1.48) | 0.76 (0.54, 1.07) | 0.76 (0.54, 1.07) | 1.00 (0.82, 1.22) | 0.82 (0.59, 1.15) |
|  | Lag1 | 1.09 (0.71, 1.69) | 0.66 (0.27, 1.65) | **0.50 (0.26, 0.95)** | **0.50 (0.26, 0.95)** | 0.96 (0.66, 1.39) | 0.72 (0.39, 1.35) |
|  | Lag2 | 1.10 (0.76, 1.59) | 0.58 (0.26, 1.29) | **0.50 (0.28, 0.89)** | **0.50 (0.28, 0.89)** | 0.93 (0.67, 1.28) | 0.77 (0.43, 1.36) |
|  | Lag3 | 0.97 (0.81, 1.16) | 0.69 (0.47, 1.01) | **0.72 (0.54, 0.95)** | **0.72 (0.54, 0.95)** | 0.94 (0.81, 1.10) | 0.91 (0.68, 1.20) |
|  | Total^1^ | 1.19 (0.39, 3.66) | 0.24 (0.02, 2.56) | **0.14 (0.02, 0.76)** | **0.14 (0.02, 0.76)** | 0.84 (0.32, 2.22) | 0.41 (0.08, 2.18) |
| Notes: ^1^ cumulative risk per 10 µg/m^3^ change in each air pollutant. Model adjusted for seasonality and long-term trend, mean temperature, relative humidity, and O_3_ or PM_2.5_. | | | | | | | |
